# Relationship Between Airway Examination with LEMON Criteria and Difficulty of Tracheal Intubation with IDS Criteria

**DOI:** 10.5812/aapm-142921

**Published:** 2024-01-16

**Authors:** Pooya Derakhshan, Nasim Nikoubakht, Mahzad Alimian, Sadaf Mohammadi

**Affiliations:** 1 Department of Anesthesiology and Pain Medicine, School of Medicine, Iran University of Medical Sciences ( IUMS ), Tehran, Iran; 2 Iran University of Medical Sciences ( IUMS ), Tehran, Iran

**Keywords:** Intubation, Airway Management, Difficult Intubation, Laryngoscopy, Mallampati, IDS, LEMON

## Abstract

**Background:**

Tracheal intubation is a common technique used to secure a patient’s airway, which is crucial in anesthesia. Successful tracheal intubation depends on various factors, including the assessment of the patient’s airway before the procedure. In recent years, scoring systems, such as LEMON (an acronym for the assessment of the airway’s appearance, identification of any dental issues, evaluation of Mallampati classification, assessment of airway obstruction, and examination of neck mobility) and intubation difficulty scale (IDS) have gained attention. This study aimed to investigate the relationship between the LEMON criteria and IDS in tracheal intubation. The goal was to provide valuable insights that can assist medical professionals in optimizing their approach to airway management by analyzing clinical data, assessing patient outcomes, and evaluating the consistency between these scoring systems.

**Methods:**

This study was based on a descriptive-analytical study involving a group of patients requiring intubation. This study examined 105 patients scheduled for elective surgeries, aged between 19 and 60 years, without specific underlying diseases, such as laryngeal cancer, temporomandibular joint stiffness, or significant tongue enlargement, and with a body mass index (BMI) below 40 kg/m². Initially, expert anesthesiologists assessed the patients using the LEMON criteria, and then the degree of intubation difficulty was measured using the IDS scoring system. Finally, these two criteria were compared.

**Results:**

In this study, there was a significant correlation between the LEMON score and the IDS score (P < 0.001). The difficult intubation group (IDS score higher than 0) had higher LEMON scores (with the highest score equal to 4) than the non-difficult intubation group (IDS score of 0) (P = 0.017). The average LEMON and IDS scores were 3.11 and 1.35, respectively. Among the participants, 96.2% had an intubation difficulty score of ≤ 5; nevertheless, 3.8% had a score of > 5. Additionally, limited neck mobility emerged as the sole independent predictor of intubation difficulty (P = 0.002, odds ratio = 6.152).

**Conclusions:**

The LEMON score is associated with difficult intubation in adult patients requiring intubation.

## 1. Background

Tracheal intubation is a vital procedure performed in various medical departments, including emergency rooms, operating rooms (1-3), and intensive care units (ICUs). This procedure involves inserting a tube into the trachea to create an artificial airway and facilitate mechanical ventilation (4). However, intubation can be challenging and might lead to complications (5), especially in patients with difficult airways (6-9).

The ability to predict difficult intubation is essential for patient safety, making scoring systems crucial in airway management. Among these systems, the LEMON criteria are used to predict difficult intubation (10); however, the intubation difficulty scale (IDS) is employed to describe the difficulty of intubation. The LEMON score evaluates five factors: External appearance, the 3-3-2 rule, the Mallampati score, airway obstruction, and neck mobility (11).

On the other hand, IDS criteria provide a standardized approach to assess and quantify the difficulty of intubation. It considers various objective factors, including the number of attempts required, the number of intubators involved, the use of peripheral airway devices, the Cormack-Lehane grade, and the need for external manipulation of the larynx. The IDS criteria offer a consistent and reliable method for assessing the complexity of the intubation procedure (12).

Several studies have explored the relationship between LEMON criteria and IDS. These investigations have demonstrated that LEMON criteria are effective predictors of difficult intubation, especially in emergency departments (2). Intubation difficulty scale criteria have also proven to be valuable tools for assessing challenging intubations in various clinical settings, such as operating rooms (13-15).

Although both the LEMON score and IDS are widely used in clinical practice, their relationship and correlation have not been thoroughly studied. Understanding the connection between these scoring systems can provide valuable insights into the predictive ability of the LEMON score and its relevance to various aspects of intubation difficulty. Therefore, this study aimed to investigate the relationship between the LEMON score and the IDS criteria in tracheal intubation.

## 2. Objectives

By analyzing data collected from a diverse population of patients undergoing intubation, this study aimed to determine whether a significant correlation exists between the LEMON score and IDS parameters. Additionally, this study will explore whether the LEMON score can reliably predict intubation failure.

## 3. Methods

This study is the result of a descriptive-analytical study among a group of patients in need of intubation at Hazrat Rasool Akram University Teaching Hospital in Tehran, Iran. After obtaining the necessary permissions and the ethics code (IR.IUMS.FMD.REC.1401.168), the researcher explained the research and education for patients, emphasizing that their information would remain confidential and the results of the research would be published anonymously. Moreover, the participants could withdraw at any time.

The sampling method of random patients was simple, and the method of determining the sample size is summarized as follows:

Desired statistical power: 80% (corresponding to a Zβ value of approximately 0.84)

Expected recurrence rate: 30% (0.30)

Acceptable margin of error: 10% (0.10)

With these values, the sample volume can be calculated using the formula previously presented:


$$n=\frac{{\left(Z_{\frac{\alpha }{2}}+Z_{\beta }\right)}^{2}\times P\times \left(1-P\right)}{{\left(P_{1}-P_{2}\right)}^{2}}$$

For a single-group study, P1 and P2 have the same recurrence rates that are denoted by P.


$$n=\frac{{\left(1.96+0.84\right)}^{2}\times 0.30\times \left(1-0.30\right)}{{\left(0.10\right)}^{2}}$$

n ≈ 89.27

Since we cannot have a fraction of a participant, we must round the sample size to the nearest whole number. Therefore, for this study, a sample size of approximately 90 participants was needed. In the present study, we enrolled and examined 105 patients between the ages of 19 and 60 years who required intubation for general anesthesia during elective surgeries. Patients meeting the following criteria were excluded from the study: Those requiring emergency intubation, patients with an American Society of Anesthesiology (ASA) classification higher than 3, patients identified as candidates for tracheal intubation with fiber optics before intubation, and patients requiring surgical airway.

Before commencing intubation and to predict the degree of intubation difficulty, the patients underwent an airway evaluation using the LEMON criteria conducted by an expert anesthesiologist. The anesthesiologist assessed the patient according to the LEMON criteria through the following steps (16):

(1) Checking externally: Reviewing the overall appearance of the patients (one point each)

Presence of a beard and mustache; obesity with a body mass index (BMI) above 25 kg/m^2^; short neck (one point for each)

(2) Long incisor teeth (buck teeth): If, when the mouth is fully closed, the upper jaw’s incisor teeth can cover the lower jaw’s incisor teeth by more than 5 mm, it receives 2 points. If they cover exactly 5 mm, it gets 1 point. Additionally, if the average height of the lower jaw’s incisor teeth is such that they cover less than 0.5 cm of the upper lip (or lower than the vermilion), indicating upper lip bite test (ULBT) class 2, it receives 1 point. However, if the incisor teeth cannot grasp the upper lip in any way, indicating ULBT class 3, it gets 2 points.

(3) Evaluating the 2: 3: 3 law: Since evaluating this law has posed challenges in various studies, we divided the criteria of this law into the following two parameters:

We instructed the patient to fully open their mouth, and if the distance between the upper and lower incisor teeth measured between 4 and 5 cm, the patient received 1 point. If the gap was less than 4 cm, they received 2 points. Then, we placed the patient’s neck in an extended position and measured the thyromental distance. If it ranged between 6 and 6.5 cm, the patient received 1 point. If it measured less than 6 cm, they received 2 points.

(4) Determining the Mallampati class: (Class 3 or 4 receives one point) (17)

The patients were seated on the bed, kept their heads in a neutral position, opened their mouths as wide as possible, and extended their tongues fully. The examiner assessed the visibility of the anatomical structures with the assistance of a light source. It is recommended to allow the patient to rest after the initial observation and then perform the examination again to ensure accuracy.

- Class 1: Clear visibility of soft and hard palates, uvula, anterior and posterior pillars, and pharynx (0 points)

- Class 2: Clear visibility of soft and hard palates, uvula, and pharynx (1 point)

- Class 3: Clear visibility of soft and hard palates and the base of the uvula (2 points)

- Class 4: Soft palate not visible (2 points)

(5) Checking for airway obstruction: The presence of foreign bodies, bleeding, clots, tumors, etc. (if present, 1 point)

(6) Assessing neck mobility: Use of a hard collar or inability to move the neck (1 point)

After the patients were scored and airway evaluation by the LEMON scale was conducted, the patients were prepared for intubation. To monitor the patient, standard devices were connected, 100% oxygen was prescribed through a mask, and the patient was encouraged to breathe normally for 3 minutes. Then, anesthetic drugs were injected for induction: Fentanyl 5 ug/kg, and after 60 seconds, 1.5 mg/kg propofol was injected intravenously. After the patient lost consciousness, 0.2 mg/kg was re-injected intravenously (18).

After 3 minutes of mask breathing and complete muscle relaxation, the patient was positioned in the sniffing position, and the intubation procedure commenced. Laryngoscopy was performed using a Macintosh blade 3 or 4, selected based on the patient’s size. Throughout the intubation process, we recorded and calculated the degree of difficulty according to the IDS criteria as follows (any additional action based on the IDS criteria was recorded as 1 point): (1) For each additional intubation attempt (redoing intubation), 1 point was assigned for each attempt; (2) each additional person required to perform separate intubation received 1 point; (3) for each additional intubation technique (e.g., repositioning the patient, changing equipment such as a tracheal tube, stylet, and blade, switching from orotracheal to nasotracheal, and using other techniques such as fiberoptic intubation) (19-21), 1 point was added for each additional technique; (4) need for increased lifting force with the laryngoscope, 1 point; (5) the application of more external laryngeal pressure to achieve a better glottis view, 1 point; (6) the adduction of vocal cords during laryngoscopy, 1 point; (7) high Cormac grade (3 and 4), 1 point.

Additionally, the degree of intubation difficulty was categorized based on the IDS score as follows: (1) A score of 0 indicated easy intubation; (2) scores ranging from 1 to 5 indicated slightly difficult intubation; (3) scores of 6 and above indicated moderate to severe intubation difficulty.

Data collection involved the completion of unique forms for each patient by the anesthesiologist, encompassing pre-intubation examinations and events during intubation. These forms contained all the necessary information for the study, including patient details (e.g., name, age, gender, and file number), physical characteristics, height, weight, Lemon criteria, and IDS criteria.

Ultimately, we compared the evaluation results and assessed the correlation between LEMON scores and the degree of intubation difficulty (IDS). Additionally, we separately examined the correlation between each of the LEMON scoring criteria and the IDS score.

Statistical analysis was conducted using SPSS software (version 18.0). The comparison of LEMON and IDS scores was performed through p-value calculations using Pearson and Kendall tests. If IDS and LEMON scores exhibited significant differences in quantitative analysis tests, they were further analyzed to calculate odds ratios (OR), 95% confidence intervals, and p-values using multiple logistic regression analysis. A significance level of P < 0.05 was considered statistically significant.

## 4. Results

In this study, we examined a total of 105 eligible patients. Among these participants, 62.9% were female; nevertheless, 37.1% were male. Regarding BMI assessment, 38.1% of the participants had a BMI below 25 kg/m^2^, indicating a healthy weight; however, 61.9% had a BMI of 25 kg/m^2^ or higher, indicating overweight or obesity.

A neck surgery scar was observed in 1% of the studied patients, and 14.3% of the investigated cases had beards and mustaches. In terms of the Mallampati classification, 48.6% of the studied patients were classified as class 2; however, 21.9% were in class 1 of the Mallampati system. Furthermore, 50.5% of the patients had a thyromental distance estimated to be more than 6.5, and 93.3% had normal neck movement without significant restriction (more than 90 degrees). In 75.2% of the examined cases, the distance between the upper and lower anterior incisor teeth was calculated to be more than 5 cm when the mouth was fully open.

Regarding intubation difficulty, 42.9% of the participants experienced an easy level of intubation, 56.2% encountered a slightly difficult level of intubation, and 1% faced moderate to severe difficulty in intubation. Demographically, there was no significant difference between different gender groups and the three categories of intubation difficulty scores (0, 1 - 5, and > 5) (P > 0.05).

The investigation did not reveal a significant correlation between the LEMON criteria and the number of intubation attempts (P = 0.09). However, a significant relationship was observed when examining the relationship between the LEMON and IDS criteria (P < 0.001). The average LEMON score among the 105 studied patients was 3.11; nevertheless, the average IDS score was 1.35. Notably, 96.2% of the participants had an intubation difficulty score of ≤ 5; nevertheless, 3.8% had a score of > 5.

**Figure 1.**
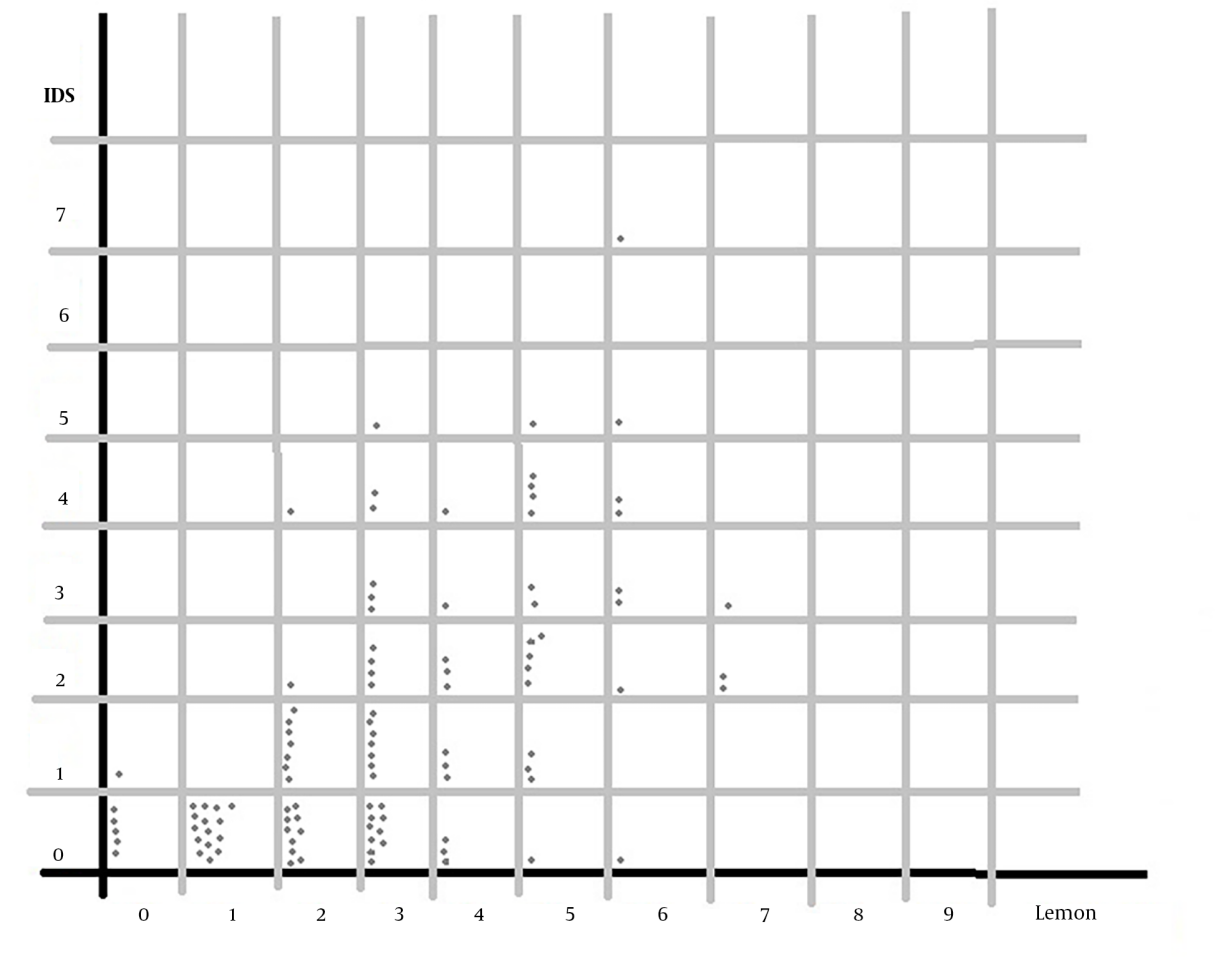
Distribution diagram of Lemon and IDS scores

In the comparison of LEMON score variables and IDS score, it was determined that neck movement restriction was the only independent predictor of intubation difficulty (P = 0.002). Other variables from LEMON and IDS were observed to be dependent (Table 1 and Figure 1). 

**Table 1.** Evaluation of Determining Variables of LEMON and Intubation Difficulty Scale (IDS) > 5

Variables	Odds Ratio	95% Confidence Interval	P-Value
**Trauma to the face**	0.983	0.320 - 3.022	0.976
**Large upper incisor teeth**	3.000	0.676 - 13.309	0.148
**Beard or mustache**	2.692	0.814 - 8.900	0.105
**Big tongue**	2.972	0.888 - 9.943	0.077
**Distance between upper and lower incisor teeth < 5**	1.827	0.662 - 5.041	0.245
**Thyroid to mental distance < 6**	0.934	0.304 - 2.867	0.905
**Signs of obstruction**	2.691	0.947 - 7.647	0.063
**Restriction of neck movement**	6.152	1.909 - 19.821	0.002

## 5. Discussion

In the conducted study, a significant correlation was observed between the LEMON and IDS scores. Among the LEMON parameters, neck mobility exhibited the highest correlation with the degree of intubation difficulty. It has already been established that the LEMON score is an effective predictor of difficult intubation in emergency departments and ICUs (22, 23).

Furthermore, the results of the present study indicated that limited neck mobility serves as an independent predictor for difficult intubation. This finding aligns with the results of other studies. When examining the relationship between the Mallampati class and intubation difficulty, it was observed that the assessment of the Mallampati class and the 3: 3: 2 rule were less accessible and less associated with intubation difficulty than cervical mobility. The problem with this challenge is that head and neck injuries often coincide with cervical spine injuries, necessitating neck immobilization, even in the absence of confirmed cervical spine injuries during trauma situations and the need for emergency intubation. During head and neck trauma or when there is suspicion of it, neck immobilization becomes crucial. However, performing a LEMON examination and determining the degree of neck mobility, which emerged as the most important parameter in this study, might not be feasible, limiting the application of this finding at the emergency level.

According to the results, the majority of study participants were female (62.9%), with 37.1% being male. In terms of BMI, 38.1% of the participants had a BMI below 25 kg/m^2^, indicating a healthy weight; nevertheless, 61.9% had a BMI of 25 kg/m^2^ or higher, indicating overweight or obesity. A significant portion of the study participants were overweight or obese, which, in addition to a high BMI score, can influence the thyromental distance, another parameter of the LEMON score. This aspect was also noted in a study by Reed et al. in which they assessed 156 patients in 2002 and 2003 using the LEMON criteria before intubation and observed a significant relationship between the likelihood of difficult intubation and a high LEMON score (P < 0.05) (23, 24).

In alignment with the findings of the study conducted by Seo et al., no differences were observed in this study concerning demographic information and gender ratio between the two groups categorized by intubation difficulty score (IDS), i.e., ≤ 4 and > 5. Seo’s study, conducted in 2012 with 305 patients in need of intubation, revealed a significant relationship between variables similar to those in the current study and IDS. Among the LEMON score parameters, ULBT exhibited the strongest correlation with IDS (25).

In the present study, a significant relationship was observed between the LEMON and Cormack criteria; nevertheless, no significant relationship was observed between the LEMON criteria and the number of intubation attempts. These findings are consistent with the results presented in a review article by Ferreira et al., who examined 9 studies related to intubation, assessing factors associated with complications and intubation difficulty. One of the factors considered was the specific airway structure of each patient (26).

Furthermore, a significant relationship was identified between the LEMON and IDS criteria in the current study. An increase in the LEMON score corresponded to an increased likelihood of difficult intubation, which was corroborated by Seo et al. and other similar studies (25). Consistent with Ji et al.’s findings, among the LEMON score variables, limited neck mobility emerged as the sole independent predictor of intubation difficulty; however, other evaluated parameters did not exhibit a significant relationship. A study conducted among 114 adult trauma patients in 2018 also detected a relationship between the modified LEMON criterion (excluding the Mallampati class parameter) and IDS (11).

### 5.1. Conclusions

This study underscores that airway examination and evaluation based on the LEMON criteria serve as reliable predictors of intubation difficulty. Neck movement limitation emerged as the most critical factor associated with intubation difficulty. In general, a patient with a LEMON score ≥ 4 might encounter difficulties during intubation. Limited neck mobility, in particular, can independently contribute to intubation challenges.

### 5.2. Limitations

Several limitations should be acknowledged in this study. Firstly, it adopted a cross-sectional design, making it impossible to establish cause-and-effect relationships. Secondly, the study exclusively focused on operating room patients, and its findings might not apply to other patient groups, such as those admitted to cardiac or surgical ICUs. Additionally, the study sample size was relatively small, highlighting the need for larger-scale investigations to validate these results further.
